# A Signaling Crosstalk between BMP9 and HGF/c-Met Regulates Mouse Adult Liver Progenitor Cell Survival

**DOI:** 10.3390/cells9030752

**Published:** 2020-03-19

**Authors:** Annalisa Addante, Cesáreo Roncero, Nerea Lazcanoiturburu, Rebeca Méndez, Laura Almalé, María García-Álvaro, Peter ten Dijke, Isabel Fabregat, Blanca Herrera, Aránzazu Sánchez

**Affiliations:** 1 Department of Biochemistry and Molecular Biology, Faculty of Pharmacy, Complutense University of Madrid (UCM), Health Research Institute of the Hospital Clínico San Carlos (IdISSC), 28040 Madrid, Spain; annalisa.addante@gmail.com (A.A.); ceronce@ucm.es (C.R.); nerelazka@gmail.com (N.L.); rebecamfilloy@gmail.com (R.M.); laura.almale@ucm.es (L.A.); mariagarciaalvaro@gmail.com (M.G.-Á.); 2 Department of Cell and Chemical Biology and Oncode Institute, Leiden University Medical Center, 2300 RC Leiden, The Netherlands; P.ten_Dijke@lumc.nl; 3 TGF-β and Cancer Group, Oncobell Program, Bellvitge Biomedical Research Institute (IDIBELL), 08907 L’Hospitalet de Llobregat, Barcelona, Spain; ifabregat@idibell.cat; 4 School of Medicine and Health Sciences, University of Barcelona, 08007 Barcelona, Spain; 5 Oncology Program, CIBEREHD, National Biomedical Research Institute on Liver and Gastrointestinal Diseases, Instituto de Salud Carlos III, 28029 Madrid, Spain

**Keywords:** BMP9, HGF, hepatic oval cell, apoptosis, signaling crosstalk, ALK1, SMAD1, p38MAPK

## Abstract

During chronic liver disease, hepatic progenitor cells (HPC, oval cells in rodents) become activated, proliferate, and differentiate into cholangiocytes and/or hepatocytes contributing to the final outcome of the regenerative process in a context-dependent fashion. Here, we analyze the crosstalk between the hepatocyte growth factor (HGF)/c-Met signaling axis, key for liver regeneration, and bone morphogenetic protein (BMP)9, a BMP family ligand that has emerged as a critical regulator of liver pathology. Our results show that HGF/c-Met signaling blocks BMP9-mediated apoptotic cell death, while it potentiates small mothers against decapentaplegic (SMAD)1 signaling triggered by BMP9 in oval cells. Interestingly, HGF-induced overactivation of SMAD1, -5, -8 requires the upregulation of TGF-β type receptor activin receptor-like kinase (ALK)1, and both ALK1 and SMAD1 are required for the counteracting effect of HGF on BMP9 apoptotic activity. On the other hand, we also prove that BMP9 triggers the activation of p38MAPK in oval cells, which drives BMP9-apoptotic cell death. Therefore, our data support a model in which BMP9 and HGF/c-Met signaling axes establish a signaling crosstalk via ALK1 that modulates the balance between the two pathways with opposing activities, SMAD1 (pro-survival) and p38 mitogen-activated protein kinases (p38MAPK; pro-apoptotic), which determines oval cell fate. These data help delineate the complex signaling network established during chronic liver injury and its impact on the oval cell regenerative response.

## 1. Introduction

Under chronic liver disease (CLD), hepatic progenitor cells (HPC, oval cells in rodents) become activated, expand into liver parenchyma and differentiate into cholangiocytes and/or hepatocytes, trying to compensate for the cellular loss and to help maintain liver homeostasis; therefore, contributing to sustain liver regeneration during hepatic disorders [1,2]. However, over the last few years, evidence supports a pro-fibrogenic role for these cells [3,4], which, together with the fact that they can also be targets of malignant conversion and become tumor-initiating cells [5], provides a rather complex picture of the precise role played by oval cells during liver damage. Certainly, appropriate regulation of these cells in the context of CLD seems to be a major determinant of their response to liver injury and subsequent outcome. Studies on the regulation of the HPCs are required to better delineate specific factors and molecular mechanisms that direct these cells towards a pro-regenerative or pro-fibrogenic status. In this regard, it is well established that hepatocyte growth factor (HGF) and its tyrosine kinase receptor, c-Met, are critical for a successful oval cell-mediated regenerative response after chronic liver damage in mice [6]. Interestingly, the absence of c-Met has profound effects in oval cells, affecting multiple cellular processes that are key for regeneration, such as proliferation, survival, differentiation, and migration [6]. In spite of the important advances achieved regarding the functions and mechanisms of action of the HGF/c-Met pathway in oval cells, it is still poorly known how HGF interplays with other factors present in the damaged liver to impact on oval cell biology. In this sense, we have just uncovered a coordinated and balanced action of HGF and transforming growth factor β (TGF-β) pathways for the regulation of the HPC/oval cell epithelial–mesenchymal transition response [7]. Indeed, HGF/c-Met signaling restrains TGF-β pro-oxidant effects, allowing cell expansion while counterbalancing the epithelial to mesenchymal switch. Our findings provide evidence that crosstalk between HGF and TGF-β pathways might be instrumental to reach an optimal HPC/oval cell regenerative potential. HGF-interplays or crosstalks with other factors remain to be identified.

Bone morphogenetic proteins (BMPs) are members of the TGF-β family, and as such, initiate signaling by binding to serine/threonine kinase receptors (type I and type II), which drives receptor complex activation and subsequent phosphorylation of small mothers against decapentaplegic (SMAD) proteins, particularly SMAD1, -5, -8. As with TGF-β, BMPs can also signal through non-SMAD pathways, such as mitogen-activated protein kinases (MAPK), among many others [8].

BMPs, and more specifically BMP9, have emerged as new regulators of liver physiology and pathology [9,10]. Recently, our group and others have elucidated a function for BMP9 in different liver pathological contexts, including acute liver damage, chronic liver fibrosis and hepatocellular carcinoma (HCC) [11,12,13,14]. We have also described that oval cells are a target of BMP9 actions. Indeed, BMP9 triggers cell growth inhibitory and pro-apoptotic effects in oval cells in vitro, effects mediated by the TGF-β type I receptor, activin receptor-like kinase-2 (ALK2) [15]. Using the 3,5-diethoxycarbonyl-1,4-dihydrocollidine (DDC) feeding model, a mouse model of cholestatic liver injury mimicking human primary sclerosing cholangitis, where HGF/c-Met signaling has proven essential [6], we have shown expression of BMP9 and activation of its downstream signaling, but more interestingly, an enhanced oval cell expansion, an improved liver function, and a decreased fibrosis in DDC-fed BMP9-KO mice [15]. Concomitantly, BMP9-KO mice showed an over-activation of HGF/c-Met signaling, providing the first hint of evidence of a potential negative crosstalk between BMP9 and HGF/c-Met pathways in the regulation of oval cells during cholestatic liver injury. These findings, together with the co-expression of HGF and BMP9 in livers from patients suffering primary sclerosing cholangitis or primary biliary cholangitis (public database GSE61256), set the grounds of the present study, aiming at elucidating a potential signaling and functional interplay between HGF and BMP9 in oval cells.

## 2. Materials and Methods

### 2.1. Reagents and Antibodies

Mouse recombinant HGF and human recombinant BMP9 were purchased from R&D Systems (Minneapolis, MN, USA). p38α/β MAPK inhibitor SB203580 was from Calbiochem (La Jolla, CA, USA). TGF-β activated kinase (TAK)1 inhibitor (5Z)-7-oxozeaenol was from Tocris Bioscience (Bristol, UK). Dulbecco’s modified Eagle’s medium (DMEM), fetal bovine serum (FBS), and trypsin-EDTA were from Gibco-Invitrogen (Barcelona, Spain). Penicillin, streptomycin, propidium iodide, DNA oligos, and buffer reagents were from Sigma-Aldrich (Tres Cantos, Madrid, Spain). HEPES and bovine serum albumin (fraction V, fatty-acid free) from Panreac AppliChem (Castellar del Valles, Barcelona, Spain). Nucleospin RNA kit (Macherey-Nagel) from Cultek (Madrid, Spain). SuperScript III RNase H Reverse Transcriptase was from Invitrogen. Oligo-dT was from Roche Diagnostics (Sant Cugat del Valles, Barcelona, Spain). ECL reagent is from Thermo-Fisher Scientific (Madrid, Spain). Caspase-3 substrate was obtained from PharMingen (San Diego, CA, USA). Primary antibodies used in this study are listed in Table S2.

### 2.2. Cell Lines and Culture Conditions

Met^flx/flx^ and Met^−/−^ oval cell lines previously generated and validated in our laboratory [16] were used as models for oval cells with functional or non-functional c-Met signaling. Cells were routinely maintained in DMEM supplemented with 10% FBS in a humidified incubator at 37 °C and a 5% CO_2_ atmosphere. Medium was replaced every three days, and cells were harvested at 80% to 90% confluence using trypsin-EDTA and replated at 1:10 dilution for maintenance. After an overnight attachment period, medium was replaced by serum-free DMEM. Cells were maintained in serum-free medium for 4–12 h prior to treatment with growth factors. Specifically, for long treatments (15 to 96 h), a short pre-starvation was enough (about 4 h), since cells were under serum-free conditions up to experiment completion. For short treatments (30 min–1 h), cells were serum-deprived for 12–15 h, to guarantee basal signaling inactivation. For HGF treatment, cells were pre-starved for 4 h, and pretreated with HGF for 12–15 h followed by BMP9 treatment (maintaining serum-free conditions all the time). SB203580 and (5Z)-7-oxozeaenol were added 30 min before addition of growth factors.

### 2.3. Analysis of Cell Number

Cells were plated and serum starved prior treatment with different factors. At various time points, cells were harvested by trypsinisation and viable cells were counted either by using a Neubauer chamber, counting 4 squares per condition or by a Casy cell counter (Roche); in both cases, each condition in triplicate.

### 2.4. Measurement of Caspase-3-like Enzymatic Activity

A fluorometric assay in the presence of Ac-DEVD-AMC as fluorogenic caspase-3 substrate was used following a previously described procedure [17]. Briefly, cells were lysed at 4 °C in 5 mM Tris/HCl, pH 8.0, 20 mM EDTA, 0.5% Triton X-100. Lysates were clarified by centrifugation at 13,000× *g* for 10 min. Reaction mixture containing 25 μL cell lysate, 325 μL assay buffer (20 mM HEPES pH 7.5, 10% glycerol, 2 mM dithiothreitol), and 20 μM caspase-3 substrate (BD Pharmingen), was incubated for 2 h at 37 °C. Proteolysis of the synthetic substrate by active caspase-3 present in the lysates releases the fluorogenic compound AMC, whose fluorescence was measured using a fluorimeter (Microplate Fluorescence Reader FL600, Bio-Tek) (excitation, 380 nm; emission, 440 nm). A unit of caspase activity is the amount of enzyme that will lead to a one unit increase in the fluorescence intensity. Protein concentration was measured using Bradford assay and results are expressed as units of activity per microgram of protein.

### 2.5. Measurement of Apoptotic Index

Measurement of apoptotic index was performed as previously described [16]. Cells were fixed with methanol:acetic acid (3:1) for 30 min at room temperature and then stained with a solution of propidium iodide (PI) (Sigma) containing 5 μg/mL PI, 0.1% Triton X-100, 0.1 M EDTA and 25 U/mL RNAse (Sigma) for 20 min at 37 °C. Finally, dishes were washed and coverslipped using Mowiol mounting medium (Sigma). Cells undergoing apoptosis were scored under inverted fluorescence microscope (Eclipse TE300, Nikon, Izasa Scientific, Alcobendas, Madrid, Spain) at high magnification (x60) following standard morphological criteria. Apoptotic indices were calculated after counting a minimum of 1000 cells per treatment in a blinded manner.

### 2.6. Protein Isolation and Western Blot Analysis

Total protein extracts from cells were prepared in IP buffer (50 mM Tris pH 7.5; 150 mM NaCl; 1% NP40; 5 mM EGTA, 5 mM EDTA) supplemented with 1 mM phenylmethylsulfonyl fluoride, 10 µg/mL aprotinin and leupeptin, 1 mM sodium orthovanadate and 20 mM sodium fluoride. Western blotting procedures were carried out as previously described [16]. Then, 30 to 80 µg of protein were separated in 10–12% acrylamide sodium dodecyl sulfate-polyacrylamide electrophoresis gels and blotted to Immobilon-P membranes (Millipore, Bedford, MA, USA). Membranes were probed with the primary antibodies diluted as indicated (Table S2) in Tris-buffered saline containing 0.1% Tween 20 and 0.5% non-fat dried milk or 0.5% bovine serum albumin according to the manufacturer’s instructions. Detection was done using the enhanced chemiluminescence (ECL) method and autoradiography.

### 2.7. RNA Isolation and Reverse Transcription–quantitative Polymerase Chain Reaction (RT–qPCR)

Total cellular RNA was isolated using the Nucleospin RNA kit (Macherey-Nagel) from Cultek (Madrid, Spain). RNA yield and purity were analyzed using a spectrophotometer (UV–visible recording spectrophotometer Specord 205, AnalytikJena, Inycom, Zaragoza, Spain). RT–qPCR was performed as described before [18]. The relative amount of target mRNA was normalized against a reference housekeeping gene (Gusb) using the 2-ΔΔCT method. Primers used in the study are presented in Table S1.

### 2.8. Gene Silencing by shRNA and siRNA

Lentivirus was produced as previously described [15,19] by co-transfecting pLKO-1 plasmids and helper plasmids pCMV-VSVG, pMDLg-RRE (gag/pol), and pRSV-REV into HEK293T cells. Cell supernatants were harvested 48 h after transfection. pLKO-1 plasmids with specific shRNAs were from Sigma (MISSION^®^ shRNA) (Sigma, Zwijndrecht, The Netherlands). Specifically, to target Activin receptor-like kinase 1 (ALK1), we used TRCN0000022540-553 (shALK1). Non-target shRNA (NT) was used as control. Cells (20% confluence) were infected for 24 h with lentiviral supernatants diluted 1:1 with normal culture medium in the presence of 8 μg/mL polybrene (Sigma), and then selected with puromycin (1 μg/mL) for at least 3 passages.

Transient SMAD1 and p38α knockdown was performed by transfection with siRNA targeting mouse SMAD1 and p38α (SMARTpool Dharmacon L-055762-00 and M-040125-01, respectively). siControl non targeting (NT) siRNA pool (Dharmacon, D-001210-03-05) was used as negative control in both cases. For siRNA transfection, we used TransITsiQuest reagent (Mirus) according to the manufacturer’s recommendation and a final siRNA concentration of 100 nM. After transfection, cells were incubated for 24 h in complete medium to allow efficient target down-regulation prior to protein harvesting or cell replating for further experiments (48 h post-transfection).

### 2.9. Transcriptional Reporter Assay

Transcriptional reporter assay was performed as previously described [20]. Briefly, cells were transiently transfected using the Plasmid pGL3 BRE Luciferase (pGL3(BRE)-luc; Promega) and, after 36 h, cells were serum starved overnight and treated with BMP9 for 6 h. Luciferase activity was quantified using the luciferase assay (Promega) with Victor luminometer (Wallac). Protein quantification was used to normalize the luciferase activity.

### 2.10. Statistical Analysis

Means ± S.E.M were used to describe each of the variables analyzed. An unpaired Student´s *t*-test or one-way ANOVA followed by the Bonferroni post hoc test were used to compare different variables between two or more experimental groups, respectively. For all analysis, *p*-values below 0.05 were considered statistically significant and were indicated in each figure. All statistical analyses were performed using GraphPad Prism 6 software (GraphPad software, San Diego, CA, USA).

## 3. Results

### 3.1. HGF/c-Met Signaling Inhibits BMP9-Triggered Apoptosis in Oval Cells while Potentiating SMAD Signaling

**Figure 1:**
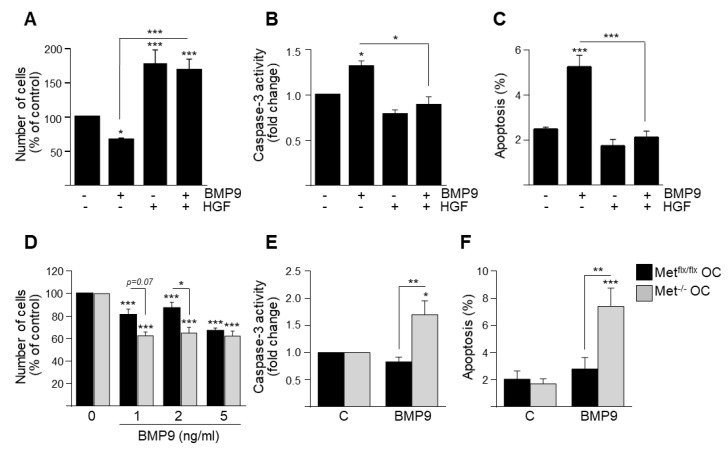
Hepatocyte growth factor (HGF)/c-Met signaling inhibits bone morphogenetic protein (BMP)9-triggered oval cell death in vitro. (**A**) HGF prevents cell loss. Met^flx/flx^ oval cells treated with BMP9 (5 ng/mL) ± HGF (40 ng/mL) were counted at day 4. Data are mean ± SEM of 4 experiments run in triplicate. (**B**,**C**) HGF impedes cell apoptosis. Met^flx/flx^ oval cells were incubated for 24 h with BMP9 (5 ng/mL) ± HGF (40 ng/mL). (**B**) Caspase-3 activity. Data are mean ± SEM of 4 experiments. (**C**) Quantification of apoptotic nuclei. Data are mean ± SEM of 3 experiments run in triplicate. (**D**) Lack of c-Met catalytic activity potentiates cell loss. Met^flx/flx^ and Met^−/−^ oval cells were treated with different concentrations of BMP9 and counted at day 4. Data are mean ± SEM of 7 experiments run in triplicate. (**E**,**F**) Lack of c-Met catalytic activity increases cell apoptosis. Met^flx/flx^ and Met^−/−^ oval cells were treated for 48 h with BMP9 (2 ng/mL). (**E**) Caspase-3 activity. Data are mean ± SEM of 3 experiments run in duplicate. (**F**) Quantification of apoptotic nuclei. Data are mean ± SEM of 3 experiments run in triplicate. Data were compared with the untreated group or as indicated * *p* < 0.05, ** *p* < 0.01, *** *p* < 0.001.

Since our previous data demonstrated that BMP9 induces apoptosis in oval cells, we first analyzed whether HGF could somehow modulate this effect. To test this, we treated Met^flx/flx^ oval cells with BMP9 in the presence of HGF and checked its effects on cell viability. HGF not only promoted oval cells proliferation by itself, as previously reported [16], but was able to prevent BMP9-induced cell loss (Figure 1A) and apoptosis (Figure 1B,C). Additionally, oval cells expressing a mutated inactive form of c-Met receptor lacking tyrosine kinase activity (Met^−/−^ oval cells) displayed enhanced sensitivity to BMP9, as evidenced by an increase in both cell loss and apoptotic index as compared to their normal counterparts (Figure 1D–F). Jointly, these results show that HGF/c-Met signaling counteracts BMP9 actions in oval cells, supporting a functional crosstalk between these two signaling pathways in oval cells.

**Figure 2:**
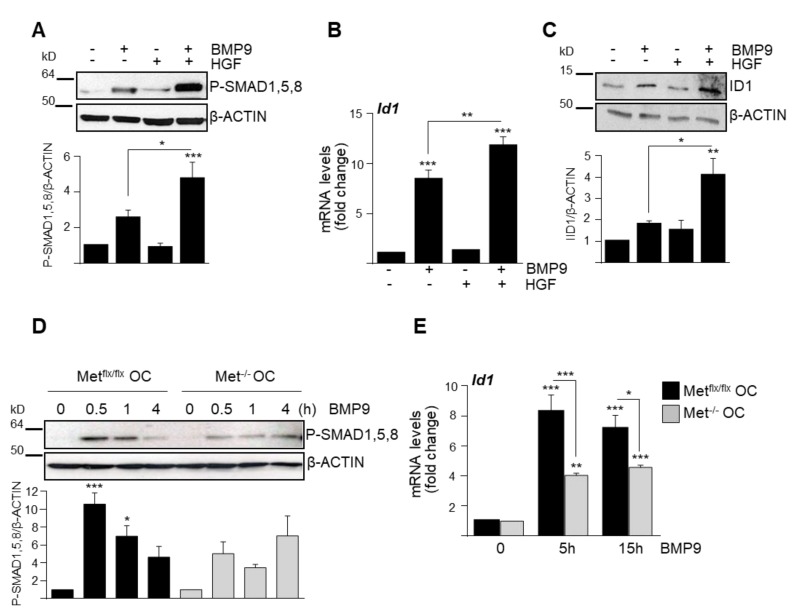
HGF/Met signaling potentiates BMP9-mediated small mothers against decapentaplegic (SMAD) activation in oval cells. (**A**) Western blot analysis of P-SMAD1, -5, -8 in Met^flx/flx^ oval cells treated for 30 min with BMP9 (2 ng/mL) ± HGF (40 ng/mL). One representative experiment is shown (upper panel). Optical density values are mean ± SEM of 8 experiments (bottom panel). (**B**,**C**) HGF potentiates BMP9-induced increase in ID1. (**B**) *Id1* levels analyzed by RT–qPCR in Met^flx/flx^ oval cells treated for 15 h with BMP9 (2 ng/mL) ± HGF (40 ng/mL). Data are mean ± SEM of 4 experiments. (**C**) Met^flx/flx^ oval cells were treated as in (**A**) and ID1 levels were analyzed by Western blot. A representative experiment of 3 is shown (upper panel). Optical density values are mean ± SEM of 3 experiments (bottom panel). **D**,**E**. Lack of c-Met catalytic activity decreases SMAD activation and SMAD target gene Id1. Western blot analysis of P-SMAD1, -5, -8 levels in Met^flx/flx^ and Met^−/−^ oval cells treated for different periods of time with BMP9 (2 ng/mL). A representative experiment is shown (upper panel). Optical density values are mean ± SEM of 4 experiments (bottom panel)**. E**. *Id1* levels analyzed by RT–qPCR in Met^flx/flx^ and Met^−/−^ oval cells treated with BMP9 (2 ng/mL) for 15 h. Data are mean ± SEM of 3 experiments. Data were compared with the untreated group or as indicated, * *p* < 0.05, ** *p* < 0.01, *** *p* < 0.001.

To directly address whether this functional crosstalk could reflect a signaling interaction between BMP9 and HGF/c-Met in oval cells, we treated Met^flx/flx^ oval cells with BMP9 in the presence or absence of HGF. Strikingly, we found that HGF potentiates BMP9-triggered SMAD1, -5, -8 signaling in oval cells (Figure 2A), resulting in a stronger induction of the SMAD transcriptional target Id1, both at the mRNA and the protein levels (Figure 2B,C). Conversely, both BMP9-induced phosphorylation of SMAD1, 5, 8 and Id1 upregulation were reduced in Met^−/−^ oval cells compared to Met^flx/flx^ oval cells (Figure 2D,E), which have an active autocrine signaling via HGF/c-Met [16]. Altogether, these data evidence that HGF/c-Met signaling positively modulates BMP9-triggered SMAD1, -5, -8 signaling in oval cells but, rather than potentiating BMP9 effects on cell death, it counteracts them.

### 3.2. BMP9 and HGF/c-Met Signaling Crosstalk in Oval Cells is Dependent on ALK1/SMAD1 Signaling Axis

Next, we aimed at characterizing the mechanism mediating BMP9-HGF signaling interaction while clarifying the apparently contradictory findings between signaling response and the resulting biological response. A plausible hypothesis was that the enhanced SMAD activation observed in the presence of HGF was a consequence of changes in the expression of BMP9 receptors in these experimental conditions. We have previously demonstrated that ALK2 and not ALK1 is the TGF-β type I receptor that mediates BMP9 signaling in oval cells [15]. We verified that the absence of a functional c-Met receptor does not alter this signaling axis, since BMP9 binds to ALK2 and BMPRII but not ALK1 in Met^−/−^ oval cells, as demonstrated by the ^125^[I]BMP-9 binding assay (Figure S1A).

**Figure 3:**
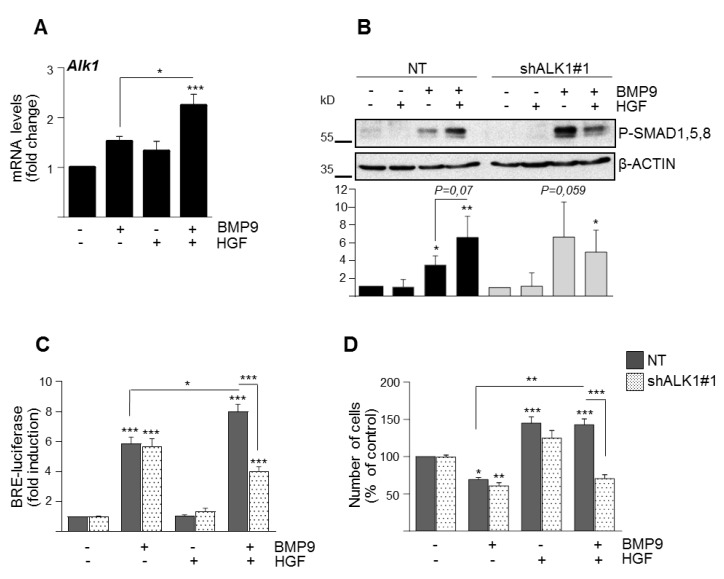
ALK1 knockdown abolishes HGF-mediated potentiation on BMP9/SMAD signaling and protective effect in oval cells. **A**. HGF and BMP9 synergistically increase Alk1 mRNA. *Alk1* levels were analyzed by RT–qPCR in Met^flx/flx^ oval cells treated for 1 h with BMP9 (2 ng/mL) ± HGF (40 ng/mL). Data are mean ± S.E.M of 3 experiments. **B**–**D.** *Alk1* knockdown reverts SMAD overactivation and oval cells survival. ALK1 knockdown oval cells (shALK1#1) and their non-targeting control (NT) oval cells were generated using Met^flx/flx^ oval cells. **B.** Western blot analysis of P-SMAD1, -5, -8 in oval cells (NT and shALK1#1) treated for 30 min with BMP9 (2 ng/mL) ± HGF (40 ng/mL). A representative experiment of 3 is shown (upper panel). Optical density values are mean ± SEM of 3 experiments (bottom panel). **C.** Luciferase activity of oval cells (NT and shALK1#1) cells transfected with pGL3(BRE)-luciferase reporter gene and treated for 15 h with BMP9 (2 ng/mL) ± HGF (40 ng/mL). Data are mean ± SEM of 3 experiments run in sextuplicate. **D**. NT and shALK1#1 oval cells treated with BMP9 (2 ng/mL) HGF ± (40 ng/mL) were counted at day 2. Data are mean ± SEM of 3 experiments run in triplicate. Data were compared with the untreated group or as indicated, * *p* < 0.05, ** *p* < 0. 01, *** *p* < 0.001.

Furthermore, by knocking down ALK2 in Met^−/−^ oval cells, we found that ALK2 mediates SMAD-dependent transcriptional response and loss of viability triggered by BMP9 in Met^−/−^ oval cells (Figure S1B–D). Nonetheless, ALK2 is not the only type I receptor expressed in oval cells. We previously showed that oval cells also express *Alk1*, although at much lower levels than *Alk2* [15]. Regarding type II receptors, oval cells express *Bmpr2*, *Acvr2a*, and *Acvr2b*. We then checked whether HGF modulates the expression of any of these receptors. Interestingly, HGF upregulates the expression of *Alk1* (Figure S2A) without affecting the expression levels of *Alk2* or the TGF-β family type II receptors *Bmpr2*, *Acvr2a,* and *Acvr2b* (Figure S2A). Furthermore, co-treatment with HGF and BMP9 resulted in a synergistic effect on *Alk1* mRNA up-regulation (Figure 3A). The expression of *Alk2* was not regulated neither by single treatment (HGF or BMP9) nor combined treatment (Figure S2B).

To directly address whether the ALK1/SMAD signaling axis could play a role in the crosstalk between BMP9 and HGF pathways in oval cells, we performed stable knockdown experiments using two different Alk1 shRNA lentiviral vectors, named as shAlk1#1 (Figure 3) and shAlk1#2 (Figure S3) and analyzed the consequences of the lack of this receptor. An 80% reduction of Alk1 mRNA levels in oval cells (Figure S3A,B) did not result in inhibition of BMP9-induced SMAD activation (the apparent increase in P-SMAD1, -5, -8 is not significant and seems to reflect nonspecific variability) nor SMAD-dependent transcriptional activity and it did not affect the BMP9 cytotoxic response in oval cells (Figure 3B–D and Figure S3). Alk1 knockdown was demonstrated at mRNA levels because both the low quality of ALK1 antibody and the low expression levels of ALK1 in oval cells make the detection of ALK1 protein decrease impossible.

**Figure 4:**
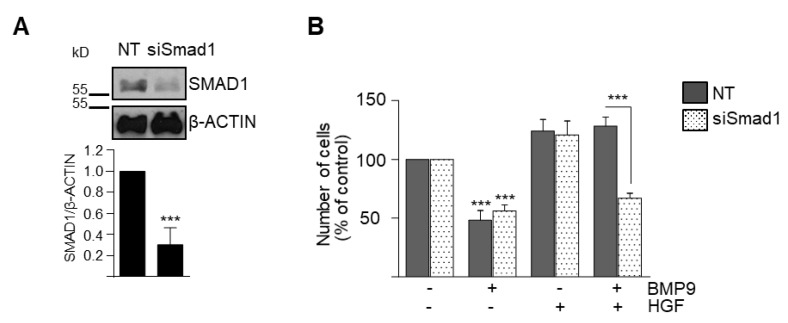
SMAD1 is required for HGF protective effect but not for BMP9-induced cell death in oval cells. Met^flx/flx^ oval cells were transiently transfected with non-targeting control siRNA (NT) or SMAD1 targeting siRNA (siSMAD1) **A**. Western blot analysis of SMAD1. A representative experiment of 3 is shown (upper panel). Optical density values are mean ± SEM of 3 experiments (bottom panel). **B.** Smad1 knockdown abolishes HGF-mediated cell survival. Oval cells (NT and siSMAD1) treated with BMP9 (2 ng/mL) ± HGF (40 ng/mL) were counted at day 2. Data are mean ± SEM of 3 experiments performed in triplicate. Data were compared with the untreated group or as indicated, *** *p* < 0.001.

However, all HGF-mediated effects, that is, amplification of BMP9-triggered SMAD1, -5, -8 phosphorylation and SMAD-dependent transcriptional activity (Figure 3B,C) and the HGF cytoprotective effect against BMP9, were lost in shALK1 oval cells (Figure 3D and Figure S3D), indicating that ALK1 is required for the HGF pro-survival effect against BMP9. On the other hand, similar to ALK1 knockdown, transient SMAD1 knockdown in oval cells using a SMAD1 siRNA did not affect BMP9-mediated decrease in oval cell number but abolished the protective effect of HGF (Figure 4A,B). Altogether, these results support that signaling via ALK1/SMAD1 is required for HGF crosstalk with BMP9, potentiating SMAD activation by BMP9 while mediating HGF protective effects against BMP9 cytotoxicity in oval cells.

### 3.3. p38MAPK Activation Mediates BMP9-Induced Apoptosis in Oval Cells

**Figure 5:**
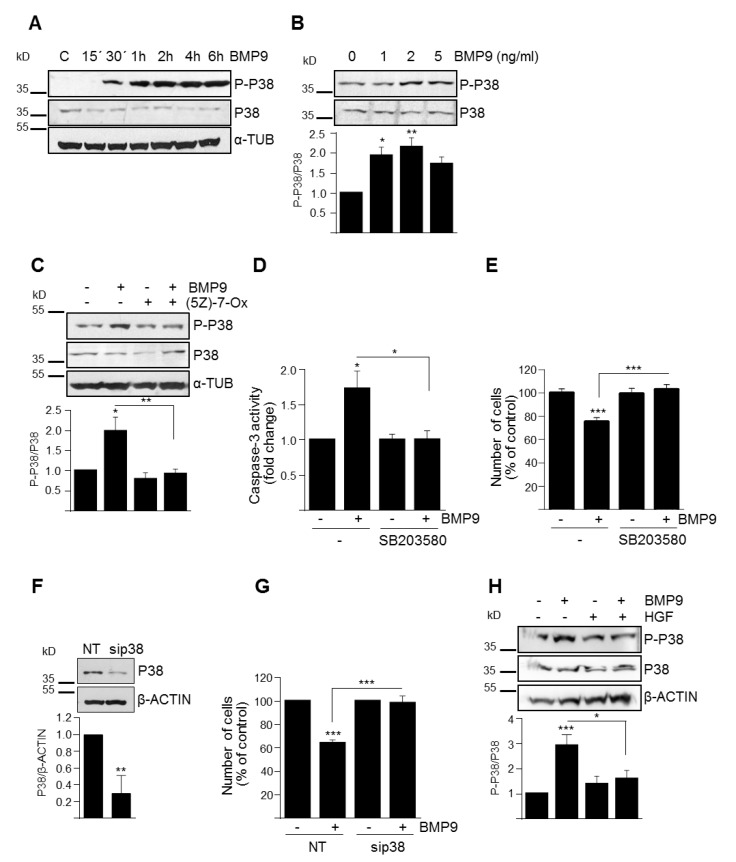
Activation of p38MAPK is required for BMP9-mediated oval cell death. **A**–**C**. Activation of p38MAPK by BMP9. Western blot analysis of P-P38 in Met^flx/flx^ oval cells treated with BMP9 (2 ng/mL) for different periods of time (**A**); or treated for 30 min with different concentrations of BMP9 (**B**) or with BMP9 (2 ng/mL) ± (5Z)-7-oxozeaenol (0.5 μM, 5Z-7-Ox) (**C**). A representive experiment of 3 is shown in each case. Optical density values are mean ± SEM of 3 experiments (bottom panels). **D**,**E.** P38MAPK inhibition impairs BMP9-induced apoptosis. Met^flx/flx^ oval cells were treated with BMP9 (2 ng/mL) ± SB203580 (10 μM). **D**. Caspase-3 activity was determined after 24 h. Data are mean ± SEM of 3 experiments run in duplicate. **E**. Cells were counted at day 2. Data are mean ± SEM. of 3 experiments run in triplicate. **F**,**G**. Knockdown of p38MAPK impairs BMP9-induced cell death. Met^flx/flx^ oval cells were transiently transfected with non-targeting control siRNA (NT) or p38 targeting siRNA (sip38). **F**. Western blot analysis of P38 levels. A representative experiment of 3 is shown (upper panel). Optical density values are mean ± SEM of 3 experiments (bottom panel). **G.** Oval cells (NT and sip38) treated with BMP9 (2 ng/mL) were counted at day 2. Data are mean ± SEM of 3 experiments run in triplicate. **H**. HGF inhibits BMP9-triggered p38MAPK activation. Western blot analysis of P-P38 in Met^flx/flx^ oval cells treated for 30 min with BMP9 (2 ng/mL) ± HGF (40 ng/mL). A representative experiment is shown (upper panel). Optical density values are mean ± SEM of 6 experiments (bottom panel). Data were compared with the untreated group or as indicated, * *p* < 0.05, ** *p* < 0.01, *** *p* < 0.001.

So far, our results indicated that SMAD1 was relevant for the HGF–BMP9 crosstalk but was not required for BMP9-induced apoptosis in oval cells. This encouraged us to clarify which non-canonical signaling pathways triggered by BMP9 in oval cells might be responsible for the BMP9 effect. Since we have previously described that activation of p38MAPK by BMP9 in HepG2 cells mediates the survival activity of BMP9 against serum withdrawal in these cells [21], we put the focus on this signaling kinase. Our results demonstrate that BMP9 activates p38MAPK in oval cells (Figure 5A,B) in an ALK2-dependent manner (Figure S4). This activation is likely mediated by transforming growth factor β-activated kinase 1 (TAK1) since it is completely abolished by pretreatment with (5Z)-7-oxozeaenol, a well-described TAK1 inhibitor [22] (Figure 5C).

To elucidate whether p38MAPK activation is responsible for the BMP9-mediated apoptotic effects, we used both a selective p38MAPK inhibitor, SB203580, and a siRNA specific for p38MAPK. Both approaches resulted in a complete abrogation of BMP9-triggered oval cell apoptotic death (Figure 5D–G). Furthermore, we found that HGF reduced the activation of p38MAPK induced by BMP9 (Figure 5H), which together with a stronger activation of p38MAPK by BMP9 in Met^−/−^ oval cells (Figure S5), cells that show a stronger apoptotic response to BMP9 (Figure 1D–F), not only confirmed a signaling crosstalk between BMP9 and HGF but further supported a role for p38MAPK as signaling mediator of the pro-apoptotic activity of BMP9 in oval cells.

## 4. Discussion

Cellular signaling crosstalk has proven relevant to provide specific and contextualized biological responses. Here, we have studied the signaling crosstalk between BMP9 and HGF pathways in hepatic oval cells and its consequences on their cell biological response. We show that HGF/c-Met axis impairs the apoptotic cell death induced by BMP9 by modulating BMP9-triggered signaling. Specifically, HGF enhances BMP9-mediated activation of SMAD canonical signaling whereas decreases p38MAPK activation, demonstrating for the first time a signaling and functional crosstalk between BMP9 and HGF/c-Met. These results are in line with studies describing a crosstalk between HGF/c-Met and other BMPs. A bidirectional regulation of expression between HGF and BMP2 exists in human osteoblasts. Thus, HGF and BMP2 upregulate each other [23,24]. Moreover, blocking c-Met signaling inhibits HGF production by BMP2 and enhances BMP2-induced osteoblast differentiation [23], indicating a biological impact of this cross-regulation. HGF also induces the expression of BMP7, as well as BMP receptors, particularly BMPR1A/ALK3, BMPR1B/ALK6, and BMPR2, in different cell types [25,26,27].

Signaling crosstalk operates in many different ways. While exploring the mechanisms of BMP9-HGF crosstalk in oval cells, we found that upregulation of ALK1 by HGF is required for the overactivation of SMAD1, -5, -8 and both ALK1 and SMAD1 are required for the counteracting effect of HGF on BMP9 apoptotic activity (Figure 3 and Figure 4). These results reveal a striking inverse correlation between the activation levels of SMAD1, -5, -8 and the apoptotic response of BMP9 in oval cells. In this regard, although activation of SMADs by TGF-β and some BMP ligands has been implicated in apoptosis induction [28,29,30,31], there are also data in the literature in favor of a pro-survival activity of SMAD1, -5, -8. In fact, Ueki et al. have demonstrated that activation of BMP-SMAD1, -5, -8 is neuroprotective, promoting the survival of retinal ganglion cells after damage *in vivo* [32]. Other examples are BMP2/SMAD1 and BMP9/SMAD1, -5 signaling, which prevented dexamethasone-induced apoptosis in the osteoblastic cell line MC3T3-E1 [33] and tumor necrosis factor (TNF)-α-induced apoptosis of pulmonary arterial endothelial cells [34], respectively. These findings, together with our results, clearly demonstrate that SMAD signaling can mediate both survival and apoptotic responses depending on many contextual factors. Our data provide solid evidence that ALK1/SMAD1 activation promotes oval cell survival upon BMP9 and HGF co-treatment, but a question arouse regarding the molecular pathways responsible for BMP9 apoptotic activity in oval cells.

By both chemical inhibition and siRNA-mediated knockdown, we demonstrate that BMP9 cytotoxic effects in oval cells depend on p38MAPK but not SMAD1 activation (Figure 4 and Figure 5), further supported by the fact that HGF reduces BMP9-induced p38MAPK activation (Figure 5), whereas impairment of c-Met signaling potentiates it (Figure S5). It is well known that BMPs, as other TGF-β family ligands, can rely on non-canonical signaling pathways to drive certain cell responses. For example, in human colon cancer cells, the anti-proliferative activity of resveratrol is partly achieved by activation of a BMP9/p38MAPK signaling pathway [35]. Additionally, we have previously demonstrated that in HepG2 cells, BMP9 activates SMAD proteins but also AKT/PI3K and p38MAPK, being p38MAPK-critical for its survival activity [21]. Recapitulating, our data show that BMP9 through ALK2 binding induces the activation of both canonical [15] and non-canonical signaling (Figure S4), being the non-canonical p38MAPK pathway the one that mediates the BMP9-induced cytotoxic effect.

The scenario changes when HGF comes into play. By inducing the expression of ALK1, the HGF/c-Met axis alters this signaling balance by enhancing SMAD1, -5, -8 phosphorylation, while reducing p38MAPK phosphorylation, overall resulting in an impairment of BMP9-induced apoptotic response. How mechanistically this signaling switch occurs remains to be defined. The simplest hypothesis would be that it all relies on a change in receptor availability. Under basal conditions, oval cells do express both ALK1 and ALK2 receptors, but ALK2 displays higher expression levels than ALK1 [15]. When ALK1 levels increase by HGF treatment, as ALK1 is a high affinity receptor for BMP9, it is conceivable that ALK1 displaced ALK2 for BMP9 binding on the oval cell surface. ALK1 and ALK2 type I receptors might indeed have different signaling properties. Supporting this, complementary actions of ALK1 and ALK2 mediate the BMP9/BMP10 effects in human aortic endothelial cells, enhancing TNF-α-induced monocyte recruitment to vascular endothelium [36]. Interestingly, a BMP6 mutant specifically defective in ALK2 binding but with normal affinity for ALK3 and ALK6 was not able to induce alkaline phosphatase expression, a target of p38MAPK [37], highlighting a key role for ALK2 on p38MAPK activation. Unfortunately, this plausible hypothesis remains to be proved and requires further experimental approaches due to difficulties in the detection of ALK1 in mouse oval cells using the available antibodies.

An additional factor to take into consideration in this signaling switch is receptor oligomerization. Previous studies on BMP signaling demonstrated that the oligomerization potential of BMP receptors is very flexible and BMP signaling outcome is different when ligand stimulates preformed receptor complexes compared to ligand-induced assembly of the same signaling complex. In this regard, ALK3 and ALK6, together with BMPRII, may be found as preformed complexes at cell membranes, preferentially activating the SMAD pathway, whereas ligand-induced signaling receptor assembly triggers the p38MAPK cascade [38,39]. Whether this might also be the case for ALK1 and/or ALK2 is still unknown but is a tempting hypothesis.

In conclusion, our data evidence an interesting signaling crosstalk between HGF and BMP9 operating in oval cells, which results in a signaling balance that determines oval cell fate. These data provide new clues to delineate the complex signaling network established during chronic liver injury. More specifically, we reveal novel mechanisms underlying the known protective activity of HGF/c-Met signaling against profibrotic and cytotoxic signals, such as BMP9, in oval cells. This is of undoubted interest from a therapeutic perspective and could help design effective therapies to reduce or revert liver damage in patients suffering from chronic liver diseases in the near future.

## Supplementary Materials

The following are available online at https://www.mdpi.com/2073-4409/9/3/752/s1. Table S1: Sequence of Primers used in quantitative reverse transcriptase-polymerase chain reaction; Table S2: List of antibodies used in Western blot analysis. Figure S1: Analysis of ALK2 as the type I receptor mediating BMP9 effects in Met^−/−^ oval cells; Figure S2: Expression of BMP9 receptors in oval cells; Figure S3: Effect of knocking down ALK1 on oval cell response; Figure S4: Effect of knocking down ALK2 on BMP9-triggered P38MAPK activation. Figure S5: Activation of p38 by BMP9 in oval cells with functional or non-functional c-Met signaling. Supplementary methods: ^125^[I]BMP-9 binding assay.
