# Supplementary material for: A Signaling Crosstalk between BMP9 and HGF/c-Met Regulates Mouse Adult Liver Progenitor Cell Survival
Source: Cells. 2020 Mar 19;9(3):752. doi: 10.3390/cells9030752 (PMC7140668; doi:10.3390/cells9030752)

Supplementary information

Supplementary table S1

Sequence of primers used in Quantitative Reverse Transcriptase-Polymerase Chain Reaction.

**Protein**	**Gene**	**Forward primer (5'-3')**	**Reverse primer (5'-3')**
ID1	*Id1*	GAGTCTGAAGTCGGGACCAC	AACACATGCCGCCTCGG
ALK1	*Acvrl1(Alk1)*	AACATCCTAGGCTTCATCGCC	AGTCATAGAGGGAGCCGTGT
ALK2	*Acvr1(Alk2)*	AGACAGCACTCTAGCGGAAC	TTCCCGACACACTCCAACAG
BMPR2	*Bmpr2*	GCAGCAGTATACAGATAGGTGA	CGCCACCGCTTAAGAGAGTAT
ActRIIA	*Acvr2a*	GCGAGAACTTCCTACGGCTT	CCTGAGTTTCTGATCTGCCA
ActRIIB	*Acvr2b*	CATTGCTGCCGAGAAACGAG	GAGGTAATCCGTGAGGGAGC
GUSB	*Gusb*	AAAATGGAGTGCGTGTTGGGTCG	CCACAGTCCGTCCAGCGCCTT

Supplementary table S2

List of antibodies used in Western blot analysis.

**Antibody Target**	**Specie**	**Dilution**	**Manufacturer (reference)**
β-ACTIN	Mouse (AC-15)	1:5000	Sigma (A5441)
ID1	Mouse (B-8)	1:500	Santa Cruz Biotechnology (sc-133104)
P38α/β	Mouse (A-12)	1:500	Santa Cruz Biotechnology (sc-7972)
P-P38	Rabbit	1:1000	Cell Signaling Technology (#9211)
P-SMAD1,5,8	Rabbit	1:1000	Cell Signaling Technology (#9516)
SMAD1	Rabbit	1:1000	Cell Signaling Technology (#9743)
α-TUBULIN	Mouse (TU-02)	1:5000	Santa Cruz Biotechnology (sc-8035)

Supplementary figure legends

Supplementary figure S1. Analysis of ALK2 as the type I receptor mediating BMP9 effects in Met$^{-/-}$ oval cells.

A. Met$^{-/-}$ oval cells were affinity-labelled with ^{125}I_BMP9 and crosslinked ligand-receptor complexes were immunoprecipitated with specific antisera as indicated and subjected to SDS-PAGE and autoradiography. **B-D**. ALK2 knockdown Met$^{-/-}$ oval cells were generated by stable infection with lentiviral vectors expressing an ALK2 targeting short hairpin RNA (shALK2; generated using TRCN0000000441) or a non-targeting short hairpin RNA (NT). **B.** *Alk2* mRNA levels were determined by RT-qPCR. Data are expressed relative to NT cells (assigned an arbitrary value of 1) and normalized using *Gusb*. **C**. Transcriptional reporter assay in NT and shALK2 oval cells treated with BMP9 (2ng/ml). Data are mean ± S.D from one representative experiment performed in sextuplicates. **D**. NT and shALK2 oval cells treated with BMP9 (2ng/ml) for 2 days were counted. Data are expressed as percentage relative to NT untreated cells and are mean ± S.E.M from 3 independent experiments performed in triplicate. Data were compared with untreated group or as indicated, ***=$p<0.001$.

Supplementary figure S2. Expression of BMP9 receptors in oval cells.

A. Oval cells were treated for 0.5 or 15 hours with HGF (40ng/ml) in 0% FBS medium. *Alk1*, *Alk2*, *Bmpr2*, *Acvr2a* and *Acvr2b* levels were analyzed by RT-qPCR and normalized to *Gusb*. Data are shown as fold change relative to untreated cells and are mean ± S.E.M from 2-3 independent experiments. **B**. Oval cells were treated for 1 hour with BMP9 (2ng/ml) in the absence or presence of HGF (40ng/ml) in 0% FBS medium. *Alk2* levels were analyzed by RT-qPCR and normalized to *Gusb*. Data are shown as fold change relative to untreated cells and are mean ± S.E.M from 2 independent experiments. Data were compared with untreated group or as indicated, *=$p<0.05$.

Supplementary figure S3. Effect of knocking down ALK1 on oval cell response.

ALK1 knockdown oval cells (shALK1#1 and shALK1#2, generated using TRCN0000022540-553 and TRCN0000231254-840, respectively) and their non-targeting control (NT) oval cells were generated by stable infection with lentiviral vectors expressing an ALK1 targeting or a non-targeting short hairpin RNA. **A-B**. *Alk1* mRNA levels were determined by RT-qPCR. Data are expressed relative to NT cells (assigned an arbitrary value of 1) (n=2). **C.** NT and shALK1 stable oval cells were transfected with pGL3(BRE)-luciferase reporter gene, then were serum starved and treated for 15 hours with BMP9 (2ng/ml) in the absence or presence of HGF (40ng/ml). Luciferase activity was normalized to cell number. Data are mean ± S.E.M. from 2 experiments run in sextuplicate and are expressed as fold change relative to untreated cells. **C**. NT and shALK1 stable oval cells were treated with BMP9 (2ng/ml) in the absence or presence of HGF (40ng/ml) in 0% FBS medium and counted at day 2. Data are mean ± S.E.M from 3 experiments performed in triplicate and are expressed as percentage of NT untreated cells. Data were compared with untreated group or as indicated, *=p<0.05; ***=p<0.001.

Supplementary figure S4. BMP9-triggered p38MAPK activation is abolished in shALK2 oval cells.

shALK2 and non-targeting control (NT) oval cells (generated as described in supplementary figure 1 legend) were treated for 30 minutes with BMP9 (2 ng/ml) in 0% FBS medium. Western blots for P-P38 and P38 were performed. A representative experiment of 3 is shown.

Supplementary figure S5. Activation of p38 by BMP9 in oval cells with functional or non-functional Met signaling.

A. $Met^{flx/flx}$ and $Met^{-/-}$ oval cells were treated for different periods of time with BMP9 (2ng/ml) in 0% FBS medium. Western blots for P-P38 were performed and β-ACTIN was used as loading control. A representative experiment of 3 performed is shown.

Supplementary methods

^{125}[I]BMP-9 binding assay

Iodination of BMP-9 was performed according to the chloramine T method and cells were subsequently affinity-labeled with the radioactive ligand as previously described (van Meeteren et al., 2008). In brief, cells were incubated on ice for 2 hours with the radioactive ligand. After incubation, cells were washed and crosslinking was performed using 54 mM disuccinimidyl suberate (DSS) and 3 mM bis(sulfosuccinimidyl)suberate (BS3 Pierce, Bleiswijk, Netherlands) for 15 minutes. Cells were washed, scraped and lysed. Lysates were boiled in sodium dodecyl sulphate (SDS) sample buffer and subjected to SDS-PAGE directly or were incubated with anti-ALK1 and anti-ALK2 antibodies overnight and immune complexes were precipitated by adding protein A Sepharose (GE Healthcare Europe). Samples were washed, boiled in SDS sample buffer and subjected to SDS-PAGE. Gels were dried and scanned with the STORM imaging system (GE Healthcare Europe).

- van Meeteren LA, Thorikay M, Bergqvist S et al. Anti-human activin receptor-like kinase 1 (ALK1) antibody attenuates bone morphogenetic protein 9 (BMP9)-induced ALK1 signaling and interferes with endothelial cell sprouting. *J Biol Chem.* 2012; 287, 18551-18561.

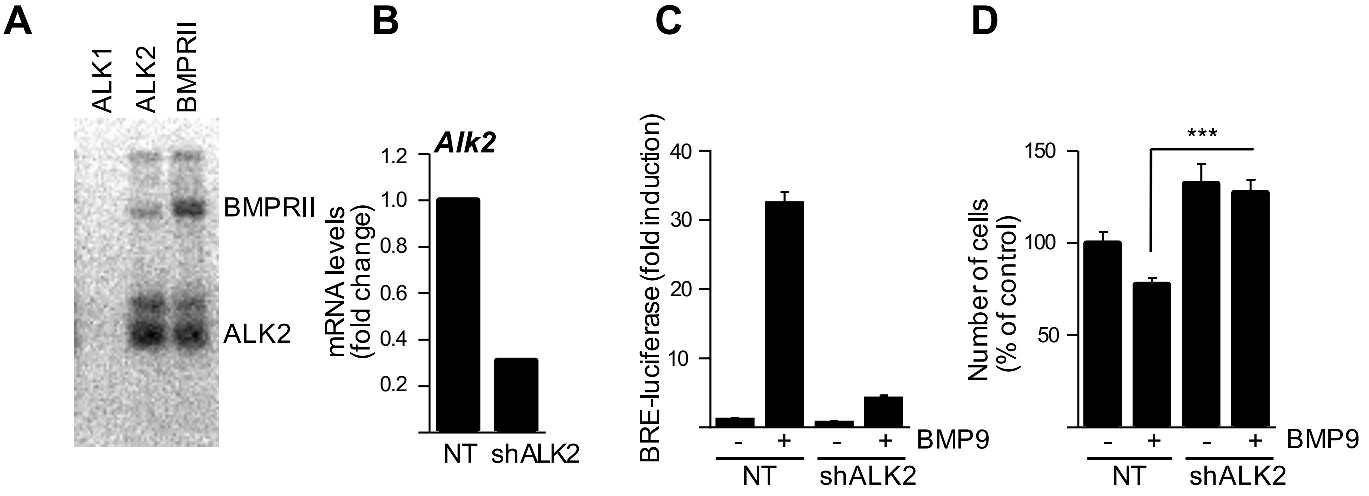
A
ALK1
ALK2
BMPRII
BMPRII
ALK2
B
Alk2
mRNA levels
(fold change)
1.2
1.0
0.8
0.6
0.4
0.2
NT
shALK2
C
BRE-luciferase (fold induction)
40
30
20
10
- + - + BMP9
NT
shALK2
D
***
Number of cells
(% of control)
150
100
50
- + - + BMP9
NT
shALK2

Supplementary Figure 2 Addante et al.

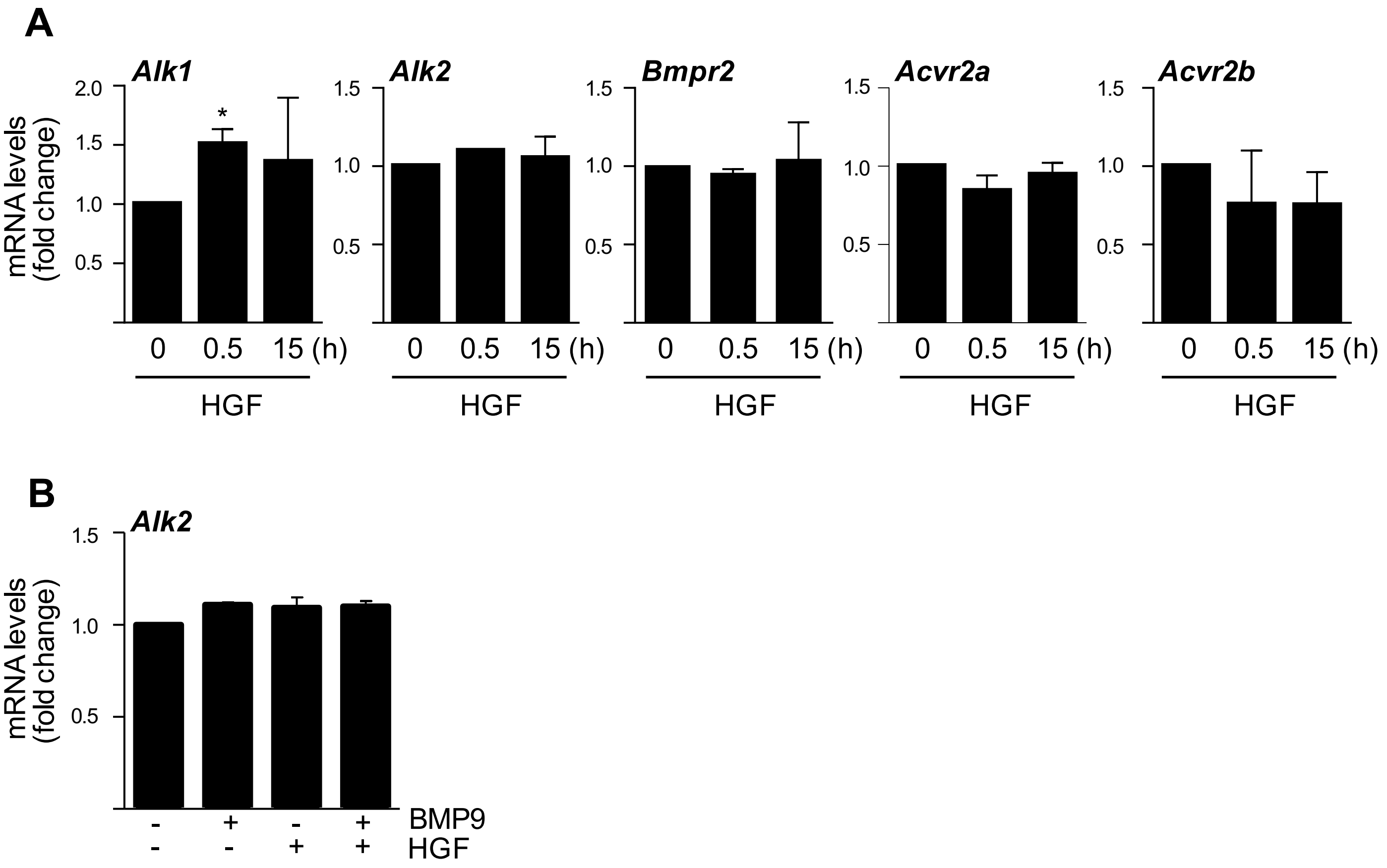

Supplementary Figure 3 Addante et al.

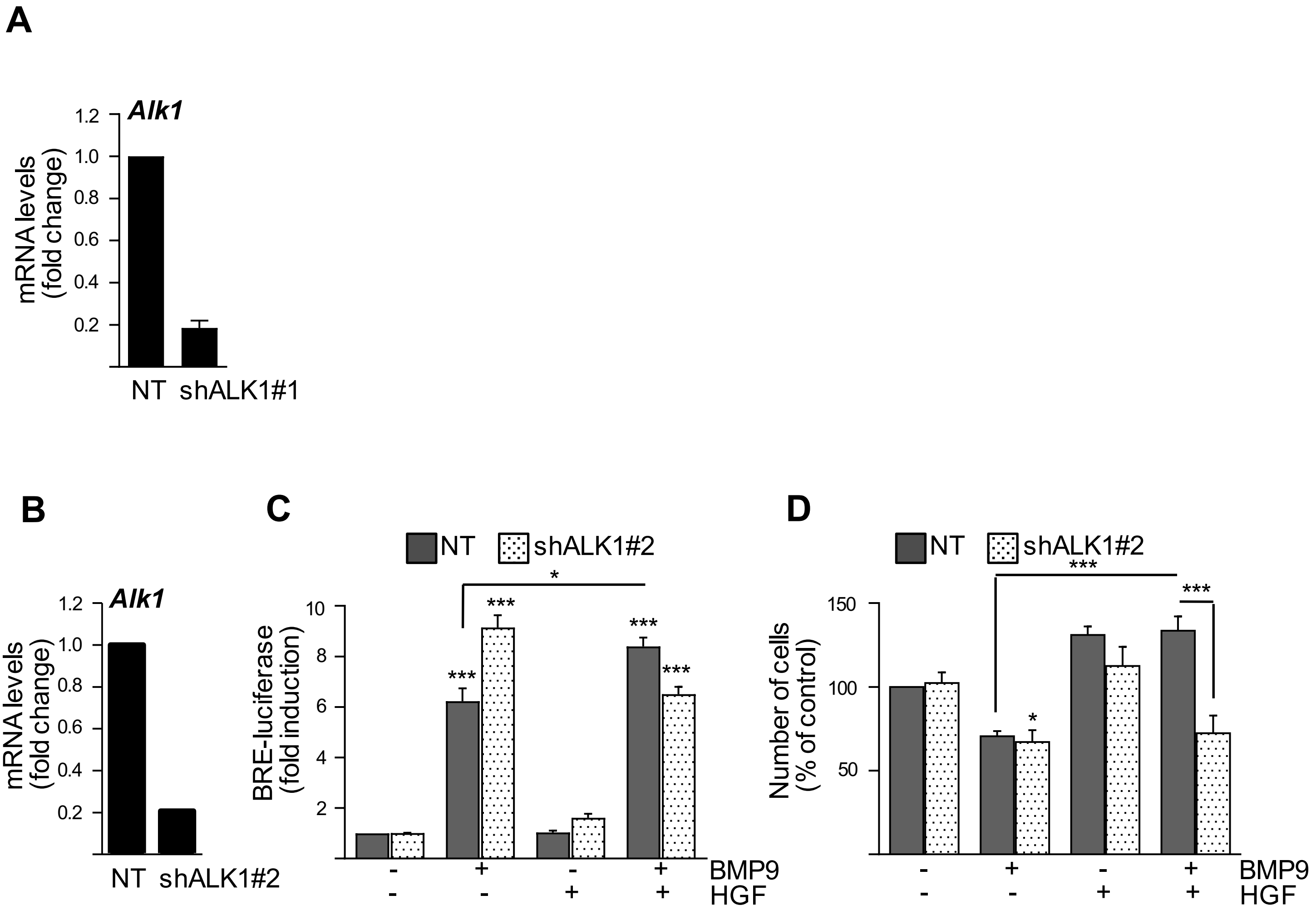

Supplementary Figure 4 Addante et al.

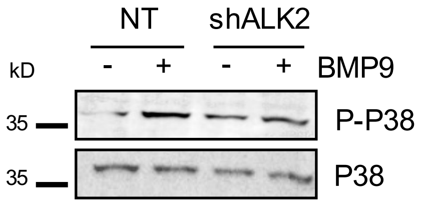

Supplementary Figure 5 Addante et al.

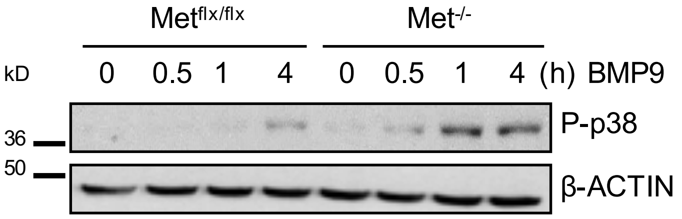

Supplement: Supplementary file 1 [file cells-09-00752-s001.pdf]
